# Supplementary material for: Impact of smoke-free ordinance strength on smoking prevalence and lung cancer incidence
Source: PLoS One. 2021 Apr 16;16(4):e0250285. doi: 10.1371/journal.pone.0250285 (PMC8051804; doi:10.1371/journal.pone.0250285)
Supplement: Attachment — Submitted filename: Response to Reviewers.docx [file pone.0250285.s002.docx]

Dr Glantz,

Thank you for your consideration and review of our manuscript and opportunity to revise and submit. We are please to submit the improved research article for your consideration in PLOS ONE. On the following pages, you will find our response to the editor and reviewer comments.

On behalf of my co-authors, I thank you for your consideration of this resubmission. We appreciate your time and look forward to your response.

Best,

Sincerely,

Ryan Nguyen, DO

University of Illinois at Chicago

840 S Wood St, 820-E CSB, MC 713
Chicago, IL 60612
Phone: 312-996-1581

Email: rnguye8@uic.edu

*Thank you for submitting your manuscript to PLOS ONE. After careful consideration, we feel that it has merit but does not fully meet PLOS ONE’s publication criteria as it currently stands. Therefore, we invite you to submit a revised version of the manuscript that addresses the points raised during the review process.*

*Please submit your revised manuscript by Apr 02 2021 11:59PM. If you will need more time than this to complete your revisions, please reply to this message or contact the journal office at plosone@plos.org. When you're ready to submit your revision, log on to https://hes32-ctp.trendmicro.com:443/wis/clicktime/v1/query?url=https%3a%2f%2fwww.editorialmanager.com%2fpone%2f&umid=31a5cdc5-6086-4765-8289-19fb71870268&auth=85c5a955287d1e42fab58bed777dfa626e5ad059-a385d6ab137389ab57cb4ed66ee6e90cdc712e27 and select the 'Submissions Needing Revision' folder to locate your manuscript file.*

*Please include the following items when submitting your revised manuscript:*

*A rebuttal letter that responds to each point raised by the academic editor and reviewer(s). You should upload this letter as a separate file labeled 'Response to Reviewers'.*

*A marked-up copy of your manuscript that highlights changes made to the original version. You should upload this as a separate file labeled 'Revised Manuscript with Track Changes'.*

*An unmarked version of your revised paper without tracked changes. You should upload this as a separate file labeled 'Manuscript'.*

*If you would like to make changes to your financial disclosure, please include your updated statement in your cover letter. Guidelines for resubmitting your figure files are available below the reviewer comments at the end of this letter.*

*If applicable, we recommend that you deposit your laboratory protocols in protocols.io to enhance the reproducibility of your results. Protocols.io assigns your protocol its own identifier (DOI) so that it can be cited independently in the future. For instructions see: http://journals.plos.org/plosone/s/submission-guidelines#loc-laboratory-protocols*

*We look forward to receiving your revised manuscript.*

*Kind regards,*

*Stanton A. Glantz*

*Academic Editor*

*PLOS ONE*

*Journal Requirements:*

*When submitting your revision, we need you to address these additional requirements.*

*1. Please ensure that your manuscript meets PLOS ONE's style requirements, including those for file naming. The PLOS ONE style templates can be found at*

*https://hes32-ctp.trendmicro.com:443/wis/clicktime/v1/query?url=https%3a%2f%2fjournals.plos.org%2fplosone%2fs%2ffile%3fid%3dwjVg%2fPLOSOne%5fformatting%5fsample%5fmain%5fbody.pdf&umid=31a5cdc5-6086-4765-8289-19fb71870268&auth=85c5a955287d1e42fab58bed777dfa626e5ad059-9e50d51f543c7be356a8c1b6a748fb3053e3adb6 and*

*https://journals.plos.org/plosone/s/file?id=ba62/PLOSOne_formatting_sample_title_authors_affiliations.pdf*

**We have updated the manuscript to meet PLOS ONE style requirements.**

*2. We noticed your Abstract has previously been published at the following publication and appears to be currently under copyright there:*

*- https://hes32-ctp.trendmicro.com:443/wis/clicktime/v1/query?url=https%3a%2f%2fascopubs.org%2fdoi%2f10.1200%2fJCO.2019.37.15%5fsuppl.6578&umid=31a5cdc5-6086-4765-8289-19fb71870268&auth=85c5a955287d1e42fab58bed777dfa626e5ad059-a18a4906472abc171a3c013e63cfae491a866e9d*

*Before we can proceed, please clarify whether the authors have received written permission from American Society of Clinical Oncology (ASCO) to publish this content specifically under the CC BY 4.0 license and upload the granted permission to the manuscript as a supporting information file. Please note that RightsLink permission forms often impose use restrictions that are incompatible with our CC BY 4.0 license, and we are therefore unable to accept these permissions.*

*For this reason, we strongly recommend contacting copyright holders with the PLOS ONE Request for Permission form. (https://hes32-ctp.trendmicro.com:443/wis/clicktime/v1/query?url=http%3a%2f%2fjournals.plos.org%2fplosone%2fs%2ffile%3fid%3d7c09%2fcontent%2dpermission%2dform.pdf&umid=31a5cdc5-6086-4765-8289-19fb71870268&auth=85c5a955287d1e42fab58bed777dfa626e5ad059-b15d5c7d04b59a9faaea6d285b99edeb8bc59587)*

**We were unable to obtain the specific CC BY 4.0 license for this abstract, as such we have modified the abstract to no longer require obtaining copyright from ASCO for the abstract being submitted in this manuscript to PLOS ONE.**

*3. In the ethics statement in the manuscript and in the online submission form, please provide additional information about the patient records/samples used in your retrospective study.*

*Specifically, please ensure that you have discussed whether all data/samples were fully anonymized before you accessed them.*

**We have updated the ethics statement to indicate all data/samples were fully anonymized before we accessed them.**

*4. We note that you have indicated that data from this study are available upon request. PLOS only allows data to be available upon request if there are legal or ethical restrictions on sharing data publicly. For information on unacceptable data access restrictions, please see https://hes32-ctp.trendmicro.com:443/wis/clicktime/v1/query?url=http%3a%2f%2fjournals.plos.org%2fplosone%2fs%2fdata%2davailability%23loc%2dunacceptable%2ddata%2daccess%2drestrictions&umid=31a5cdc5-6086-4765-8289-19fb71870268&auth=85c5a955287d1e42fab58bed777dfa626e5ad059-6bd67d2e302e59d8d1d9f13691bd4da8c206a046.*

*In your revised cover letter, please address the following prompts:*

*a) If there are ethical or legal restrictions on sharing a de-identified data set, please explain them in detail (e.g., data contain potentially identifying or sensitive patient information) and who has imposed them (e.g., an ethics committee). Please also provide contact information for a data access committee, ethics committee, or other institutional body to which data requests may be sent.*

*b) If there are no restrictions, please upload the minimal anonymized data set necessary to replicate your study findings as either Supporting Information files or to a stable, public repository and provide us with the relevant URLs, DOIs, or accession numbers. Please see https://hes32-ctp.trendmicro.com:443/wis/clicktime/v1/query?url=http%3a%2f%2fwww.bmj.com%2fcontent%2f340%2fbmj.c181.long&umid=31a5cdc5-6086-4765-8289-19fb71870268&auth=85c5a955287d1e42fab58bed777dfa626e5ad059-f8d40643eb43b63efe44ba4fb1211cd57abaadeb for guidelines on how to de-identify and prepare clinical data for publication. For a list of acceptable repositories, please see https://hes32-ctp.trendmicro.com:443/wis/clicktime/v1/query?url=http%3a%2f%2fjournals.plos.org%2fplosone%2fs%2fdata%2davailability%23loc%2drecommended%2drepositories&umid=31a5cdc5-6086-4765-8289-19fb71870268&auth=85c5a955287d1e42fab58bed777dfa626e5ad059-4f8ea64c6c40d88f0cb5822e7e32889eb4ec41ef.*

*We will update your Data Availability statement on your behalf to reflect the information you provide.*

**We have provided a minimal anonymized data set.**

*5. Thank you for stating the following financial disclosure: 'No'*

*At this time, please address the following queries:*

*Please clarify the sources of funding (financial or material support) for your study. List the grants or organizations that supported your study, including funding received from your institution.*

*State what role the funders took in the study. If the funders had no role in your study, please state: “The funders had no role in study design, data collection and analysis, decision to publish, or preparation of the manuscript.”*

*If any authors received a salary from any of your funders, please state which authors and which funders.*

*If you did not receive any funding for this study, please state: “The authors received no specific funding for this work.”*

*Please include your amended statements within your cover letter; we will change the online submission form on your behalf.*

**We have updated our statement to “The authors received no specific funding for this work.”**

*6. Please include a separate caption for each figure in your manuscript.*

**We have included a separate caption for each figure in our manuscript.**

*7. Please ensure that you refer to Figures 2 and 3 in your text as, if accepted, production will need this reference to link the reader to the figure.*

**We have referred to figures 2 and 3 in the text.**

*8. Please include your tables as part of your main manuscript and remove the individual files. Please note that supplementary tables should be uploaded as separate "supporting information" files.*

**We have included the tables as part of the manuscript. Per the email from Jazmin Toth (PONE-D-21-01837R1) on 3/15/21, I have re-included the tables as separate files.**

*[Note: HTML markup is below. Please do not edit.]*

*Reviewers' comments:*

*Reviewer's Responses to Questions*

*Comments to the Author*

*1. Is the manuscript technically sound, and do the data support the conclusions?*

*The manuscript must describe a technically sound piece of scientific research with data that supports the conclusions. Experiments must have been conducted rigorously, with appropriate controls, replication, and sample sizes. The conclusions must be drawn appropriately based on the data presented.*

*Reviewer #1: Yes*

*2. Has the statistical analysis been performed appropriately and rigorously?*

*Reviewer #1: Yes*

*3. Have the authors made all data underlying the findings in their manuscript fully available?*

*The PLOS Data policy requires authors to make all data underlying the findings described in their manuscript fully available without restriction, with rare exception (please refer to the Data Availability Statement in the manuscript PDF file). The data should be provided as part of the manuscript or its supporting information, or deposited to a public repository. For example, in addition to summary statistics, the data points behind means, medians and variance measures should be available. If there are restrictions on publicly sharing data—e.g. participant privacy or use of data from a third party—those must be specified.*

*Reviewer #1: No*

*4. Is the manuscript presented in an intelligible fashion and written in standard English?*

*PLOS ONE does not copyedit accepted manuscripts, so the language in submitted articles must be clear, correct, and unambiguous. Any typographical or grammatical errors should be corrected at revision, so please note any specific errors here.*

*Reviewer #1: Yes*

*5. Review Comments to the Author*

*Please use the space provided to explain your answers to the questions above. You may also include additional comments for the author, including concerns about dual publication, research ethics, or publication ethics. (Please upload your review as an attachment if it exceeds 20,000 characters)*

*Reviewer #1: This paper is well undertaken and well written replication of results in Indiana reported earlier by Hahn and colleagues for Kentucky. This earlier study is appropriately referenced*

*In the first sentence of the last paragraph of the introduction, the authors claim that SFOs are one of the most effective public policy means of decreasing smoking prevalence and SHS exposure. While the reference they use is appropriate, it is not sufficient for such a strong statement. Suggest the authors add a reference to an official report such as:*

*IARC Handbooks of Cancer Prevention, Tobacco Control, Vol. 13: Evaluating the effectiveness of smoke-free policies (2009: Lyon, France)*

*Another relevant reference for this would be Azagba S, Shan L, Latham K. County Smoke-Free Laws and Cigarette Smoking Among U.S. Adults, 1995-2015. Am J Prev Med. 2020 Jan;58(1):97-106.*

**We have updated this citation with the two references provided, thank you for your suggestion.**

*6. PLOS authors have the option to publish the peer review history of their article (what does this mean?). If published, this will include your full peer review and any attached files.*

*If you choose “no”, your identity will remain anonymous but your review may still be made public.*

*Do you want your identity to be public for this peer review? For information about this choice, including consent withdrawal, please see our Privacy Policy.*

*Reviewer #1: Yes: John P Pierce*

*[NOTE: If reviewer comments were submitted as an attachment file, they will be attached to this email and accessible via the submission site. Please log into your account, locate the manuscript record, and check for the action link "View Attachments". If this link does not appear, there are no attachment files.]*

*While revising your submission, please upload your figure files to the Preflight Analysis and Conversion Engine (PACE) digital diagnostic tool, https://pacev2.apexcovantage.com/. PACE helps ensure that figures meet PLOS requirements. To use PACE, you must first register as a user. Registration is free. Then, login and navigate to the UPLOAD tab, where you will find detailed instructions on how to use the tool. If you encounter any issues or have any questions when using PACE, please email PLOS at figures@plos.org. Please note that Supporting Information files do not need this step.*
